# Tooth-whitening treatment with potassium sodium tartrate: a non-invasive method that preserves enamel integrity

**DOI:** 10.1038/s41405-026-00405-4

**Published:** 2026-02-03

**Authors:** Angelina Ivanova, Valeriia Buzova

**Affiliations:** 1 SkyLab AG, Route de la Corniche 6, 1066 Epalinges, Lausanne, Switzerland; 2 School of Physical and Chemical Sciences, Queen Mary University of London, London, UK

**Keywords:** Special care dentistry, Tooth whitening

## Abstract

**Objectives:**

Tooth-whitening treatments in modern dentistry often led to enamel demineralization and sensitivity. This study explored potassium sodium tartrate, a piezoelectric material which employs a piezoelectricity effect to gently remove stains, as a non-invasive alternative to traditional peroxide-based whitening methods which chemically oxidize extrinsic stains causing enamel demineralization and sensitivity. Specifically, the research measured stain removal, enamel integrity, addressing common drawbacks of peroxide treatments.

**Materials and methods:**

The research was focused on two in vitro studies assessed sodium potassium tartrate efficacy for whitening and enamel preservation compared to carbamide peroxide. In the first experiment bovine enamel blocks (n = 10/group) were stained and treated with prototype toothpastes (2% potassium sodium tartrate, 2% carbamide peroxide, base-only control, deionized water) via simulated brushing followed by a cumulative 3.5-hour immersion to model extended action. Stain Removal Index (SRI%) and Surface Microhardness Recovery (%SMHR) were measured. In the second experiment Stained human enamel (n = 8-10/group) was treated with commercial-type toothpastes, including those containing 2% potassium sodium tartrate, over simulated 1-week and 1-month periods. Whitening was quantified as VITA® Bleachedguide shade changes. Instrument calibration and ethical sample sourcing were as per standardized protocols.

**Results:**

In the first experiment the potassium sodium tartrate and carbamide peroxide groups showed statistically equivalent stain removal (SRI%: 30.06 ± 7.08 vs. 30.02 ± 6.58). However, carbamide peroxide significantly reduced enamel microhardness (%SMHR: -15.80 ± 4.38), whereas potassium sodium tartrate preserved it (0.08 ± 4.06), similar to non-whitening controls. In the second experiment after one month, the potassium sodium tartrate formulation with fluoride achieved comparable shade improvement (4.76 ± 1.51 shades) to the peroxide-fluoride control (4.38 ± 0.58 shades).

**Conclusions:**

In vitro results indicate potassium sodium tartrate could provide an effective and enamel-safe alternative to peroxide-based whitening, meriting further clinical investigation.

## Introduction

The emphasis on physical appearance and maintenance of oral health in contemporary culture has resulted in tooth-whitening treatment becoming an intrinsic facet of dentistry [1]. Tooth-whitening treatment has witnessed a surge in popularity and is poised for continued expansion owing to advances in technological innovation and heightened public awareness [2]. In dental literature, a distinction is often made between the general term “tooth whitening,” which encompasses any process that makes teeth appear whiter (including mechanical stain removal), and the more specific term “tooth bleaching,” which refers strictly to chemical treatments using oxidizing agents (e.g., peroxides) to lighten the intrinsic color of dental hard tissues. Several types of tooth-whitening methods are available at present, and each method is characterized by its own distinct attributes [3].

Abrasive whitening is the most popular method for the removal of extrinsic staining in daily routine. Most manufacturers of cosmetic toothpaste incorporate abrasives into toothpaste formulations as insoluble particulate components [4]. The physical hardness of abrasives is superior to that of dental stains [5], and this property facilitates the mechanical removal of superficial stains on tooth surfaces. Abrasive agents predominantly target extrinsic stains and are confined to areas accessible via toothbrushing. Thus, they cannot alter tooth color, and their effect is limited with respect to malposed teeth, interdental spaces, and gingival regions [5, 6].

Peroxide-based whitening is the most powerful method used for whitening and removal of extrinsic stains. Contemporary tooth-whitening systems predominantly contain peroxides, specifically hydrogen peroxide and carbamide peroxide, as whitening agents [7]. Peroxide-based whitening relies on the infiltration of oxygen into the dental tissues, which catalyzes the degradation of sizeable pigment molecules, rendering them shorter and devoid of coloration [8]. This pivotal mechanism culminates in the elimination of the yellowish tint. Consumables, such as food, tinted beverages, and tobacco, must be avoided for a minimum of 24 hours post-treatment [9–11]. Peroxide-based whitening is contraindicated for individuals below the age of 14 years, pregnant and lactating women, and individuals with enlarged and progressive demineralization of the enamel. Moreover, protracted use of whitening agents may compromise dentin integrity [12], increase tooth sensitivity [13], and lead to demineralization of the enamel [14].

Enzymatic whitening is a safer and more feasible alternative for cleaning and partial whitening [15, 16]. The ability of enzymes to remove extrinsic staining is attributed to the incorporation of organic compounds harboring chromophores (the molecular segment responsible for coloration) within the acquired dental plaque [6]. In addition to inducing dissociation of the bonds between proteins and color-contributing compounds, proteolytic enzymes (e.g., papain and bromelain) also induce hydrolytic cleavage and the subsequent removal of the biofilm formed on tooth surfaces over time. However, this method is associated with potential adverse effects, such as abdominal discomfort, perspiration, and constipation, resulting from allergic reactions caused by exposure to oral enzymes [16].

Acid-based whitening is a growing field in dentistry. Various products containing acidic constituents, such as citric acid, tartaric acid, and ascorbic acid, are frequently used in tooth-whitening treatment [17–21]. However, there are some limitations to their application. Acids disintegrate calcium reservoirs within the dental tissues [22], thereby eliminating their yellowish hues. Thus, the application of acids can have unfavorable consequences, such as heightened tooth sensitivity, enamel erosion, and mucosal irritation [21], and its recurrent use may incite allergic reactions in the oral mucosa [21].

The formulation of innovative and more efficacious tooth-whitening modalities without deleterious effects on the enamel has been an avenue of exploration in recent years. One such notable avenue of exploration is the use of piezoelectric materials. The bound charges are balanced by shielding charges on the surface of a polarized piezoelectric material [22], rendering the materials electrically neutral [23]. The amplitude of polarization is reduced owing to the compressive stress (i.e., negative deformation) resulting from the piezoelectric effect, leading to the redistribution of charge carriers and release of additional shielding charges from the surface. Excess charges dissipate in the solution and become free charges that combine with water molecules to form reactive species such as •${OH}$ and •${O}_{2}^{-}$ [24]. The bound charges are minimized under maximum mechanical stress, and excess shielding charges are released until the material reaches a new electrostatic balance [25]. A decrease in the applied force (i.e., unloading) leads to an increase in polarization, causing charges to be adsorbed from the surrounding electrolyte to balance the bound charges induced by the piezoelectric effect [26]. Electrolyte charges with polarity opposite to that of the adsorbed charges participate in redox reactions, generating •${OH}$ and •${O}_{2}^{-}$ reactive species. Thus, when subjected to periodic loads in an electrolyte environment, a piezoelectric material continuously produces •${OH}$ and •${O}_{2}^{-}$ reactive species, akin to photocatalysis under light stimuli [26–29]. The piezo catalytic effect of these materials can be incorporated into toothpaste formulations via the replacement of conventional abrasive components found in toothpaste with piezoelectric particles [30] or adding them as an additional whitening agent. In this application, mechanical stress from brushing polarizes the piezoelectric particles, driving redox reactions that generate localized reactive oxygen species (ROS). These ROS, such as hydroxyl radicals (•${OH}$) and superoxide anions (•${O}_{2}^{-}$)then oxidize and break down the organic chromophores responsible for extrinsic stains, effectively converting brushing energy into a targeted chemical cleaning action. In stark contrast to conventional whitening agents, such as peroxides or organic acids, piezoelectric materials have a non-destructive and innocuous effect on dental enamel [31]. Potassium sodium tartrate, a piezoelectric material, provides a potential solution to this issue. The piezoelectric effect enables mechanical stress to generate reactive oxygen species, facilitating whitening without the damaging oxidative process of peroxide-based treatments. This study investigates its efficacy in whitening while preserving enamel.

The incorporation of materials such as potassium sodium tartrate (${KNa}{C}_{4}{H}_{4}{O}_{6}\cdot 4{H}_{2}O$) [32], in toothpaste formulations and teeth whitening systems has garnered significant attention due its nature of origin [31] owing to their improved effects on the oral mucosa and enamel. Potassium sodium tartrate is a versatile compound used historically in piezoelectric devices, [33]. It is derived from Argol, a winemaking byproduct. It is neutralized with caustic soda to pH 8, decolorized with activated charcoal, and filtered. The solution is then evaporated and slowly cooled to crystallize the tartrate [34]. Potassium sodium tartrate has an acceptable daily intake (ADI) of 30 mg/kg body weight, established by the Scientific Committee for Food in 1990. Studies indicate that metabolism varies by species, with rats absorbing more than humans. No toxic effects or genotoxicity related to tartaric acid or its salts have been observed [35]. Therefore, this study aimed to evaluate potassium sodium tartrate’s tooth-whitening efficacy compared to peroxide-based treatments; to assess its impact on enamel preservation; to examine its clinical performance in an innovative toothpaste formulation. The null hypothesis (H₀) posits that the incorporation of potassium sodium tartrate into toothpaste formulations has no significant effect on the whitening of enamel or the preservation of enamel integrity compared to carbamide peroxide or formulations without whitening agents. Conversely, the alternate hypothesis (H₁) suggests that the incorporation of potassium sodium tartrate into toothpaste formulations significantly improves enamel whitening while maintaining enamel integrity, outperforming carbamide peroxide and formulations without whitening agents [36–40].

## Materials and methods

### Materials

Potassium sodium tartrate (CAS 304-59-6), carbamide peroxide (CAS 124-43-6) were purchased from Sigma-Aldrich (Sigma Chemical Co. Ltd., St. Louis, MO, USA) and used as active ingredients for tooth-whitening toothpastes. The substances had a purity level of at least 99.0%. In addition, solvents, thickeners, preservatives, surfactants, pH buffers, humectants, and abrasives were also used for the formulation of cosmetic- and pharmaceutical-grade toothpaste formulations. Potassium sodium tartrate is a well-characterized piezoelectric material, and its fundamental piezoelectric charge generation coefficients are established in the literature. Therefore, in this study, we utilized its inherent piezoelectric properties without performing separate calibration of its charge coefficients, focusing instead on the evaluation of its functional whitening efficacy and enamel safety in a toothpaste formulation.

For the preparation of the stain-uptake solution, hydrochloric acid (HCl), sodium carbonate, and phytic acid were procured from Sigma-Aldrich (UK). The staining solution was prepared using the following ingredients: Tryptic Soy Broth (TSB, Cat. No. T8907, Sigma-Aldrich, UK), PG Tips pure tea granules, Nescafe Original instant coffee, mucin type II (CAS 84082-64-4, Sigma-Aldrich, UK), and ferric chloride (CAS 7705-08-0, Sigma-Aldrich, UK).

### General experimental procedures

#### Tooth sample preparation and ethics

The in vitro tests were performed under contract by Intertek CRS (UK). The study design and data interpretation were the sole responsibility of the authors. Bovine incisors (for Experiment 1) and human molars extracted for orthodontic reasons (for Experiment 2) were used. The bovine incisors were sourced and prepared by our contract research partner, Intertek Clinical Research Services (UK). As documented in the provided Intertek CRS Safety Statement and their official DEFRA (Animal & Plant Health Agency) Registration, these teeth are a by-product of the food-processing industry, obtained exclusively from licensed slaughterhouses under Category 3 animal by-product regulations (material from animals slaughtered for human consumption). No animals were sacrificed for this research. Human teeth were obtained under HTA-licensed protocols (License No. 12169, holder: ITS Testing Services UK Ltd.) with informed consent. Human enamel blocks were prepared from extracted molars. The samples were sourced, processed, and utilized in full compliance with ethical and regulatory standards. All teeth were obtained post-extraction from consenting adult patients at UK dental clinics under a Human Tissue Authority-licensed protocol (HTA License No. 12169). Prior to donation, patients received written and verbal information about the research, and their anonymized verbal consent was obtained. No patient-identifiable information was transferred. To ensure safety and declassify the material under the Human Tissue Act, all collected teeth were rendered acellular and sterile via gamma irradiation prior to sample preparation. All samples were stored in 0.1% thymol solution at 4 °C until use.

Enamel blocks (5 × 5 mm) were cut using a precision saw, embedded in resin (epoxy for bovine, PMMA for human) exposing only the enamel surface, and sequentially polished to a mirror finish using aluminum oxide slurry (0.3 µm). Before staining, all blocks underwent a standardized etch procedure (1 min in 1% HCl, 30 s in saturated Na₂CO₃, 1 min in 1% phytic acid) to enhance stain uptake, followed by thorough rinsing with deionized water.

#### Staining protocol

A staining broth was prepared by dissolving TSB (30 g/L) in deionized water at 50°C, followed by the addition of instant coffee (3.4 g/L), tea infusion (from 5 g/L of tea leaves), mucin (2.5 g/L), and ferric chloride (0.05 g/L). Enamel blocks were mounted on a custom rig and subjected to cyclic immersion in the heated (50°C) staining broth at 1 rpm for 96 hours to achieve consistent, heavy extrinsic staining.

#### Colorimetry and microhardness measurements

Color measurements (CIE L, a, b*) were performed using a calibrated Konica Minolta CM-700d spectrophotometer (D65 illuminant, 10° observer, SCI mode, 3 mm aperture). The instrument was calibrated before each measurement session using the manufacturer’s proprietary white calibration cap and zero calibration box to ensure accuracy and reproducibility of the CIE L*a*b* readings. Each sample was measured at four orientations, and the mean value was calculated.

Surface microhardness (SMH) was measured using a Knoop indenter (Buehler MicroMet 5103) under a 50 g load applied for 10 s. The tester was calibrated prior to the measurement series using certified reference blocks with known hardness values to verify the accuracy of the applied 50 g load and the indentation measurement system. Five indentations were made per sample, and the average was recorded. Percentage Surface Microhardness Recovery (%SMHR) was calculated as:

$${{SMHR}},\, \% \,= 	 \,({{{SMH}}}_{{{Post}}-{{treatment}}}\,-\,{{{SMH}}}_{{{Post}}-{{demineralisation}}})/\\ 	 ({{{SMH}}}_{{{Baseline}}}\,-\,{{{SMH}}}_{{{Post}}-{{demineralisation}}}\,)\,\times \,{{100}} \%$$

#### Brushing simulation

Toothpaste slurries were prepared by mixing 1 part toothpaste with 1.6 parts deionized water (w/w) using a high-shear mixer. Brushing simulations were performed using automated brushing machines (Martindale M235D for Exp. 1; V8 Cross-Brushing Machine for Exp. 2) with a flat-head brush and a 150 g load. The machines were calibrated to ensure a consistent vertical load of 150 g and standardized brushing stroke parameters throughout all experiments. The brushing time was standardized to simulate one week (52.5 s) or one month (210 s) of clinical exposure per single tooth, based on recommended oral hygiene regimens. Following brushing, the samples were immersed in the assigned toothpaste slurry for 3.5 hours. We acknowledge that this continuous immersion represents a controlled in vitro model rather than a direct simulation of the dynamic in vivo oral environment, where clearance via saliva, swallowing, and spitting would rapidly reduce active concentration. The objective of this protocol was not to replicate clinical pharmacokinetics but to establish a standardized and severe test condition that eliminates clearance as a variable, allowing for a direct comparative assessment of the intrinsic, extended stain-removal potential of the different active agents (potassium sodium tartrate vs. carbamide peroxide). This cumulative immersion period (equivalent to the sum of 14 theoretical 15-minute post-brushing contact episodes over two weeks) was designed to provide a discriminating model for sustained efficacy, creating a reproducible framework to evaluate the maximum whitening effect under conditions of prolonged contact. While a protocol involving multiple brief treatments could more closely approximate individual clinical events, the single immersion was chosen for practical reproducibility and to avoid compounding variability from repeated handling and slurry renewal. This approach tests the agents’ capacity for sustained action under equivalent conditions.

#### Statistical analysis

Statistical analysis was performed using Minitab software (v18 or 21). Data normality and homogeneity of variance were assessed. For multiple group comparisons, one-way or two-way ANOVA followed by Tukey’s post-hoc test was applied. Significance was set at *p* < 0.05. Data are presented as mean ± standard deviation.

### Experiment 1: In vitro study to compare the ability of toothpaste with active ingredients and a negative control to whiten teeth and to assess the effect on enamel structure

The experimental design utilized forty bovine enamel blocks, which were prepared, polished, and subjected to a robust extrinsic staining protocol as detailed in the general procedures. Following staining, the blocks were stratified into four treatment groups of ten samples each, ensuring equivalent initial stain levels across groups based on their post-staining L* color values. The treatment groups were as follows: Group 1.1 received a toothpaste containing 2% carbamide peroxide (positive control), Group 1.2 was treated with a prototype toothpaste containing 2% potassium sodium tartrate, Group 1.3 received a toothpaste without active ingredients, and Group 1.4 was treated with deionized water as a negative control. For this comparative study, three experimental toothpaste formulations and one negative control were prepared on an identical, cosmetic-grade base (containing standard abrasives, humectants, surfactants, thickeners, and pH buffers). The formulations differed solely in their active component: (1) Base (solvents, thickeners, preservatives, surfactants, pH buffers, humectants, and abrasives) + 2.0% potassium sodium tartrate, (2) Base + 2.0% carbamide peroxide (carbamide peroxide, positive control), and (3) Base only (without an active whitening agent).

Each group underwent a controlled treatment cycle designed to simulate one week of clinical use. This cycle consisted of a standardized mechanical brushing simulation with the respective toothpaste slurry, immediately followed by a prolonged static immersion phase in the same slurry. This combined approach aimed to replicate both the abrasive and the chemical contact phases of real-world toothpaste application. The key outcome measures were assessed at three time points: at baseline, after staining, and after the complete treatment cycle. Quantitative analysis included the calculation of the total color change (ΔE) and the Stain Removal Index (SRI%), derived from spectrophotometric measurements. Enamel surface microhardness was meticulously measured using a Knoop indenter, and the percentage of Surface Microhardness Recovery (%SMHR) was calculated for each sample to quantify the restorative effect on enamel strength. Furthermore, representative samples from each group were examined using scanning electron microscopy (SEM) at high magnification (2000x and 4000x) to allow for a qualitative, direct assessment of surface topography and the detection of any morphological alterations, such as excessive erosion or pitting.

### Eхperiment 2: In vitro study to compare the ability of three toothpaste formulations versus a negative control to whiten teeth in terms of VITA® bleachedguide shades whiter

This study employed forty stained human enamel blocks, prepared and etched to ensure consistent and heavy extrinsic staining that reached the darkest shades of the VITA® Bleachedguide scale. After staining, the blocks were stratified into four treatment groups matched for baseline stain level: Group 2.1 (2% carbamide peroxide toothpaste, positive control, n = 10), Group 2.2 (2% potassium sodium tartrate toothpaste, n = 10), Group 2.3 (2% potassium sodium tartrate with 1455 ppm fluoride toothpaste, n = 10), and Group 2.4 (deionized water, negative control, n = 9, with one sample excluded for not meeting staining criteria), as outlined in Table 1. This study evaluated three commercially relevant toothpaste formulations: (i) a positive control toothpaste containing 2% carbamide peroxide and 1455 ppm fluoride; (ii) a marketed toothpaste (Biomed MOLECULAR WHITENING) with 2% potassium sodium tartrate; and (iii) a variant of the latter toothpaste with 2% potassium sodium tartrate and added fluoride (1455 ppm). Deionized water served as the negative control. A critical foundational step for this experiment was the prior establishment of a precise correlation between instrumental color difference (ΔE) and clinical shade steps. As shown in Fig. S1, a calibration curve was created by measuring each tab of a physical VITA® Bleachedguide (Fig. S2) with the spectrophotometer. This curve confirmed a strong linear relationship where a ΔE change of approximately 1.0 unit corresponded to a one-shade improvement on the guide, enabling the direct conversion of spectrophotometric data into clinically meaningful “shades whiter” values.

**Table 1:** Test products

Test Product	N	Treatment Group
Toothpaste with 2% peroxide carbamide and 1445ppm Fluoride	10	1
Biomed MOLECULAR WHITENING Enamel strengthening with 2% potassium sodium tartrate	10	2
Biomed MOLECULAR WHITENING Enamel strengthening with 2% potassium sodium tartrate and 1445ppm Fluoride	10	3
Deionised Water, Scientific Laboratory Supplies, Product Code: CHE3876, Batch Number: 911808, Expiry: 30/05/2025	9	4

The treatment protocol involved brushing simulations equivalent to two distinct time points: one week and one cumulative month of use per single tooth. Color measurements (L*, a*, b*) were taken at baseline, after staining, and after each simulated treatment interval using the calibrated spectrophotometer under standardized conditions. The total color change (ΔE) for each sample at each time point was calculated and subsequently converted into the number of VITA® Bleachedguide shades whiter using the pre-determined correlation. Descriptive statistics were calculated for the shade change achieved by each formulation, and the data were analyzed using General Linear Model ANOVA followed by Tukey’s test to identify statistically significant differences between the treatment groups at the one-week and one-month endpoints. This methodology provided a clear, standardized, and clinically relevant direct comparison of the whitening efficacy progression over time between the test formulations and controls.

## Results

### In vitro study to compare the ability of toothpaste with active ingredients and a negative control to whiten teeth and to assess the effect on enamel structure

#### Stain removal effect on bovine teeth

The stain removal values of the three toothpaste formulations were significantly higher than that of the negative control (Table 2). All three toothpaste formulations achieved significantly greater percentages of stain removal than the negative control, indicating that they were effective in removing the extrinsic staining. The formulations containing 2.0% Potassium Sodium Tartrate and 2.0% peroxide carbamide achieved the largest mean percentages of stain removal (30.06% and 30.02%, respectively) after the equivalent of one week’s worth of brushing and exposures to the test articles. The formulation without whitening agents achieved a mean percentage of stain removal value of 15.61%, indicating moderate efficacy. Statistically significant differences were observed between the percentages of stain removal achieved by the two toothpaste formulations containing potassium sodium tartrate and peroxide carbamide and those of the formulation without whitening agents. Deionised water achieved the lowest mean percentage of stain removal value, with a slightly negative mean percentage of stain removal value (-2.0%).

**Table 2:** Post-treatment mean percentage of stain removal for different toothpaste formulations.

Toothpaste Formulation	*N*	Mean Percentage of Stain Removal with SD, %	Statistical Comparison Groupings^a^	Pairwise *p*-value
Toothpaste with 2.0% of potassium sodium tartrate	10	30.06 ± 7.08	A	-
Toothpaste with 2.0% of peroxide carbamide (active control)	10	30.02 ± 6.58	A	-
Toothpaste without whitening agents (negative control)	10	15.61 ± 5.46	B	<0.001 (vs. A)
Deionised water	9	−2.25 ± 3.76	C	<0.001 (vs. A and B)

^a^Means that do not share letters are significantly different. Grouping Information Using the Tukey Method and 95% Confidence. *P* < 0.05 is a significant difference.

Tables 3–5 show the cropped images of the enamel samples in each treatment group at baseline, after staining, and after brushing. This study sought to replicate conditions that are conducive to tooth surface staining using aggressive pigments, such as coffee, tea, and ferric chloride, which augment the infiltration of colouring agents. Visual assessment and colorimetric measurements revealed that the toothpaste formulation with potassium sodium tartrate exhibited the highest efficacy in eliminating pigmented stains from the enamel when exposed with the toothpaste formulation with 2.0% potassium sodium tartrate and the toothpaste formulation containing 2.0% carbamide peroxide.

**Table 3:** (a-b): Cropped images of bovine enamel samples before the application of the staining solution, showing their natural colour.

Treatment	Baseline samples colour photographs
Toothpaste with 2.0% potassium sodium tartrate	
Toothpaste with 2.0% peroxide carbamide	
Toothpaste without whitening agents	
Deionised water	

Each treatment group contains 10 samples.

**Table 4:** Cropped images of bovine enamel samples after application of staining solution.

Treatment	Staining samples photographs
Toothpaste with 2.0% potassium sodium tartrate	
Toothpaste with 2.0% peroxide carbamide	
Toothpaste without whitening agents	
Deionised water	

(a) 10 stained samples for treatment with 2% sodium potassium tartrate toothpaste; (b) 10 stained samples for treatment with 2% peroxide carbamide toothpaste; (c) 10 stained samples for treatment with toothpaste without whitening agent; (d) 9 stained samples for treatment with deionised water.

**Table 5:** Cropped images of bovine enamel samples after 1 week’s worth brushing.

Treatment	Brushed samples photographs
Toothpaste with 2.0% potassium sodium tartrate	
Toothpaste with 2.0% Peroxide Carbamide	
Toothpaste without Whitening Agents	
Deionised Water	

(a) 10 treated samples with 2% sodium potassium tartrate toothpaste slurry; (b) 10 samples treated with 2% peroxide carbamide toothpaste slurry; (c) 10 samples treated with toothpaste without whitening agent slurry; (d) 9 samples treated with deionised water.

#### Surface Microhardness assessment with the surface microhardness recovery (%SMHR)

The mean percentages of the surface microhardness recovery values achieved by the test products are presented in Table 6. The formulation without whitening agents and the formulation containing 2.0% potassium sodium tartrate achieved the highest mean SMH of 1.74% and 0.08%, respectively, after the equivalent of 1-week’s worth of brushing and exposure to the test articles (Table 6). A 15.80% decrease in the microhardness of enamel was observed following treatment with the formulation containing 2.0% peroxide carbamide, indicating a destructive effect on the enamel and further acute demineralisation resulting in painful sensitivity. The formulation containing potassium sodium tartrate and the formulation without whitening agents achieved significantly greater SMHR values than the formulation containing carbamide peroxide. No statistically significant differences were observed between the SMHR values of the formulation containing potassium sodium tartrate, the formulation without whitening agents, and deionised water.

**Table 6:** In vitro surface microhardness recovery (SMHR) results of bovine enamel samples.

Treatment	*N*	Mean percentage of surface microhardness recovery with SD (SMHR ± SD), %		Statistical comparison groupings^a^	Pairwise p-value (vs. Peroxide Carbamide	
Deionised water	9	−0.44 ± 6.80			A	*p* < 0.001
Toothpaste without whitening agents	10	1.74 ± 5.49			A	*p* < 0.001
Toothpaste with 2.0% potassium sodium tartrate	10	0.08 ± 4.06			A	*p* < 0.001
Toothpaste with 2.0% peroxide carbamide	10	−15.80 ± 4.38			B	-

Data are presented as mean SMHR (%) ± SD.

^a^Means that do not share letters are significantly different. Grouping Information Using the Tukey Method and 95% Confidence. *P* < 0.05 is a significant difference.

#### SEM analysis after the application of toothpastes with and without whitening agents

**Fig. 1:**
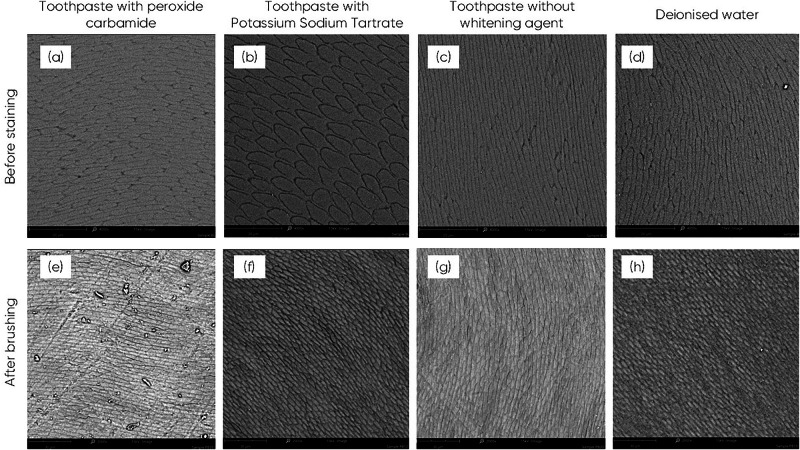
SEM images of enamel samples. Comparison of the SEM images of control enamel samples before staining (**a**–**d**) and after treatment with the formulations: **e** toothpaste with peroxide carbamide, **f** toothpaste with potassium sodium tartrate, **g** toothpaste without whitening agents, **h** deionised water. Magnification 4000x. Scale 20 µm. Executed using SEM (scanning electron microscope).

The enamel samples underwent SEM analysis before staining and after brushing for the evaluation of their morphology to assess the surface morphology of various specimens. Sound enamel displayed characteristic hydroxyapatite crystallites arranged in a typical keyhole configuration (Fig. 1a–d). Enamel samples treated with the formulation containing potassium sodium tartrate or the formulation without whitening agents did not show any changes in enamel surface integrity. In contrast, enamel samples treated with the formulation containing 2.0 weight % of peroxide carbamide showed interprismatic dissolution (Fig. 1e) when compared with untreated samples (Fig. 1a). The hydroxyapatite structure on the enamel surface of the sample treated with deionised water (Fig. 1h) was nearly identical to that of the control (Fig. 1d).

### In vitro study to compare the ability of three toothpaste formulations versus a negative control to whiten teeth in terms of VITA® Bleachedguide shades whiter

The mean post-brushing number of VITA® Bleachedguide shade whiter values achieved by the toothpaste formulations and the negative control (Deionised water) after the equivalent of 1 week’s worth and 1 month’s worth of brushing for a single tooth are shown in Table 7.

**Table 7:** Post-brushing mean number of VITA® Bleachedguide shades whiter after 1 week and 1 month of brushing with different treatments.

Time Point	Treatment Group	N	Mean VITA® Shades Whiter with SD	Statistical comparison groupings^a^	Pairwise p-values
1 Week	2.1	8	3.07 ± 0.80	A	1 vs. 2 < 0.001, 1 vs. 3 < 0.001, 1 vs. 4 < 0.001
	2.2	8	2.59 ± 0.64	AB	2 vs. 3: 0.092, 2 vs. 4: < 0.001
	2.3	8	1.82 ± 0.46	B	3 vs. 4: < 0.001
	2.4	8	0.34 ± 0.07	C	-
1 Month	2.3	8	4.76 ± 1.51	A	3 vs. 1: 0.307, 3 vs. 2: 0.013, 3 vs. 4: < 0.001
	2.1	8	4.38 ± 0.58	A	1 vs. 2: 0.435, 1 vs. 4: < 0.001
	2.2	8	3.54 + 1.03	A B	2 vs. 4: < 0.001
	2.4	8	0.55 + 0.18	C	-

^a^Means that do not share letters are significantly different. Grouping Information using the Tukey Method and 95% Confidence. *P* < 0.05 is a significant difference.

After the equivalent of 1 week’s worth of brushing for a single tooth, the toothpaste formulation coded Toothpaste with peroxide carbomide achieved the highest mean number of VITA® Bleachedguide shades whiter, achieving a mean VITA® Bleachedguide shade whiter value of 3.07 shades whiter.

The statistical analysis of the 1-week data showed the toothpaste formulation coded Toothpaste with peroxide carbomide achieved statistically significantly more VITA® Bleachedguide shades whiter than the treatments coded Biomed MOLECULAR WHITENING Enamel strengthening with Fluoride and Biomed MOLECULAR WHITENING Enamel strengthening, which have achieved the next highest mean number of VITA® Bleachedguide shades whiter, achieving mean VITA® Bleachedguide shade whiter values of 2,59 and 1,82 respectively. (Table 7).

After the equivalent of 1 month’s worth of brushing for a single tooth, the toothpaste formulations coded Biomed MOLECULAR WHITENING Enamel strengthening with Fluoride, Toothpaste with peroxide carbomide achieved the highest mean numbers of VITA® Bleachedguide shades whiter, achieving mean VITA® Bleachedguide shade whiter values of 4.76 and 4.38 respectively.

The statistical analysis of the 1-month data showed the toothpaste formulations coded Biomed MOLECULAR WHITENING Enamel strengthening, Biomed MOLECULAR WHITENING Enamel strengthening with Fluoride, Toothpaste with peroxide carbomide, achieved statistically significantly more VITA® Bleachedguide shades whiter than the treatment coded Deionised Water (0,55) (Table 7).

After the equivalent of 1 month’s worth of brushing for a single tooth, the toothpaste formulation coded Biomed MOLECULAR WHITENING Enamel strengthening achieved the next highest mean number of VITA® Bleachedguide shades whiter, achieving mean VITA® Bleachedguide shade whiter value 3.54.

A photograph of the enamel samples after staining and after the equivalent of 1 week’s and 1 month’s worth of brushing for a single tooth is provided in Figures S3–S6.

All toothpaste formulations assessed in this study were effective at removing extrinsic staining, achieving on average between 2 and 3 VITA® Bleachedguide shades whiter after the equivalent of 1 week’s worth of brushing for a single tooth and between 3 and 5 VITA® Bleachedguide shades whiter after the equivalent of 1 month’s worth of brushing for a single tooth.

After the equivalent of 1 week’s worth of brushing for a single tooth the toothpaste formulation coded Toothpaste with peroxide carbamide achieved the highest mean number of VITA® Bleachedguide shades whiter.

After the equivalent of 1 month’s worth of brushing for a single tooth the toothpaste formulations coded Biomed MOLECULAR WHITENING Enamel strengthening with Fluoride, Toothpaste with peroxide carbamide achieved the highest mean numbers of VITA® Bleachedguide shades whiter (4.76 + 1.31 and 4.38 + 0.58 respectively).

## Discussion

The effect of tooth-whitening procedures on the enamel is of paramount importance. Conventional tooth-whitening products involving hydrogen peroxide which serve as active agents in cosmetic products such as toothpaste and mouth rinses. No 1223/2009 of the European Parliament and the Council dated November 30, 2009, has permitted the use of peroxides at concentrations of up to 2.0% of the pure compound in toothpaste formulations [41]. Hydrogen peroxide generate unstable reactive oxygen species during their decomposition in water [42], which subsequently target and break down organic pigment molecules adhering to the tooth surfaces, effectively oxidising the staining compounds. However, this oxidative process may also result in the weakening of enamel and dentin [42].

The piezoelectric effect, initially discovered by Pierre Curie and Jacques Curie in 1880 [43], is characterised by the accumulation of electric charge in specific solid materials with noncentrosymmetric structures when subjected to mechanical stress. A material capable of exciting and releasing reactive oxygen species can serve as an effective tooth-whitening agent. This mechanism, unlike that of peroxide carbamides, leads to the release of microdoses of reactive oxygen species, thereby enabling a more precise and targeted enamel bleaching process while minimising potential harm.

Our null hypothesis was rejected as Potassium sodium tartrate had whitening effects on teeth. Results indicate that the incorporation of Potassium Sodium Tartrate in whitening toothpastes is effective, as the findings are statistically significant when compared to those obtained using deionized water. After 1 week of usage, teeth exhibit an average whitening of approximately 2 shades. Although this outcome is less pronounced than that achieved with toothpaste containing carbamide peroxide, it is important to note that, based on the mentioned results, it is less harmful to the enamel surface.

When brushing with Potassium Sodium Tartrate toothpaste for 1 month, the whitening results are superior to those achieved with toothpaste containing carbamide peroxide, but this comparison holds true only for fluoride toothpaste. However, Potassium Sodium Tartrate toothpaste without fluoride also demonstrated impressive whitening results, achieving an improvement of 3.5 shades in just 1 month.

Research indicates that the formulation containing potassium sodium tartrate maintains the structural integrity of enamel without affecting its microhardness. This observation highlights a significant benefit of utilizing potassium sodium tartrate as a bleaching agent: its capacity to protect enamel while achieving similar levels of whitening effectiveness. The core finding of this study is that potassium sodium tartrate provides the whitening effect preserving enamel integrity: in a direct comparison, a 2% potassium sodium tartrate formulation achieved stain removal efficacy (30.06% ± 7.08) statistically equivalent to the market benchmark, 2% carbamide peroxide (30.02% ± 6.58), while completely avoiding the significant enamel softening (%SMHR = -15.80% ± 4.38) induced by the peroxide treatment. This divergence in safety, despite equivalent efficacy, underscores a critical advantage: whereas a conventional “treat-and-repair” paradigm relies on remineralizing agents to counteract peroxide-induced demineralization, potassium sodium tartrate enables a “preserve-while-treating” approach. By achieving the primary aesthetic goal without initiating the cycle of enamel damage, it may reduce the long-term need for compensatory therapies and offers a fundamentally gentler pathway for sustained tooth whitening and health. This effectiveness was confirmed through in vitro evaluations of the final products, Biomed MOLECULAR WHITENING Enamel Strengthening and Biomed MOLECULAR WHITENING Enamel Strengthening with Fluoride. Crucially, this study directly underscores the practical potential of potassium sodium tartrate as a viable active ingredient in commercial toothpaste formulations. The significant whitening efficacy and enamel-safe profile demonstrated in our first experiment, which utilized prototype pastes with a standardized base, confirm its fundamental suitability for inclusion in oral care products. This translational potential is further validated by the performance of the commercially developed BIOMED toothpastes in the second experiment.

It is widely believed that enamel lightening products can lead to increased dentinal sensitivity [9]. Our data confirm that the novel toothpaste has a positive effect on Dentinal hypersensitivity due to gentle lightening preserving enamel integrity (that was confirmed in in vitro study) and additional enamel strengthening due to adding HAP to the composition. This leads to increased customer satisfaction with enamel lightening product by ensuring strong yet gentle effect which meets customers expectation. Therefore, this research demonstrates that potassium sodium tartrate fundamentally challenges the common compromise in over-the-counter whitening, offering benchmark-level efficacy coupled with an enamel-safe profile. In conclusion, potassium sodium tartrate offers an innovative approach to tooth whitening, combining efficacy with enamel protection. The successful application of this piezoelectric mechanism is defined by two specific prerequisites: (1) the formulation must contain piezoelectric particles (e.g., potassium sodium tartrate) as the active catalytic agent, and (2) it requires mechanical activation via brushing to provide the stress necessary to polarize the particles and trigger the stain-removing redox reaction. A key limitation of potassium sodium tartrate is its reliance on mechanical action to activate the piezoelectric effect, which is essential for its whitening mechanism. This restricts its application to formulations that involve brushing, such as toothpastes, tooth powders, or tablet-based toothpastes, as the friction generated by brushing is necessary to induce the piezoelectric response. Consequently, potassium sodium tartrate cannot be utilized in gel-based systems, whitening strips, or mouth rinses. This limitation highlights the need for further exploration of potassium sodium tartrate’s applicability in various delivery systems. Future studies could explore its broader applications and long-term benefits in oral care formulations.

## Conclusion

The findings of this in vitro investigation demonstrate that potassium sodium tartrate, leveraging the piezoelectric effect, presents a novel mechanism for tooth whitening. When incorporated into toothpaste formulations, it achieved a level of stain removal statistically equivalent to the current benchmark agent, 2% carbamide peroxide. Crucially, the potassium sodium tartrate-based treatments maintained enamel surface microhardness, in stark contrast to the significant demineralization caused by the peroxide control. This combination of comparable efficacy and superior enamel safety observed in controlled laboratory settings underscores the potential of potassium sodium tartrate to fulfill the need for effective yet gentle whitening agents.

The successful translation of these results into prototype and commercial-type (BIOMED) toothpaste formulations confirms its practical applicability. However, the efficacy of this mechanism is contingent upon mechanical activation via brushing, defining its primary application in dentifrices. Therefore, based on the present in vitro evidence, potassium sodium tartrate emerges as a highly promising candidate for safer tooth whitening. Subsequent research, including fully powered randomized clinical trials, is essential to validate these findings in vivo, assess long-term efficacy and safety, and explore its potential in broader oral care applications.

## Supplementary information

Supplemental 1

## Data Availability

The data presented in this study are available on request from the corresponding author.
