# Supplementary material for: Tooth-whitening treatment with potassium sodium tartrate: a non-invasive method that preserves enamel integrity
Source: BDJ Open. 2026 Feb 3;12:14. doi: 10.1038/s41405-026-00405-4 (PMC12868650; doi:10.1038/s41405-026-00405-4)
Supplement: Supplementary file 1 — Supplemental 1 [file 41405_2026_405_MOESM1_ESM.docx]

Supplementary material

Article

Tooth-Whitening Treatment with Potassium Sodium Tartrate: A Non-Invasive Method That Preserves Enamel Integrity

Angelina Ivanova^1,2^*, Valeriia Buzova^1^

1. SkyLab AG, Route de la Corniche 6, 1066 Epalinges, Lausanne, Switzerland; [ivanova@skylaboratory.ch](mailto:ivanova@skylaboratory.ch)
2. School of Physical and Chemical Sciences, Queen Mary University of London, London, UK

***** Correspondence: ivanova@skylaboratory.ch

Supplementary figures


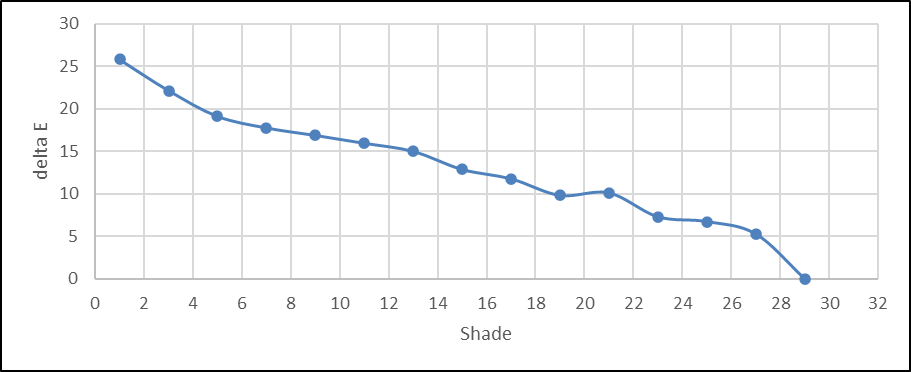

**Figure S1**. A correlation graph for the conversion of the total color change (Delta E) to the number of Bleachedguide VITA® shades.


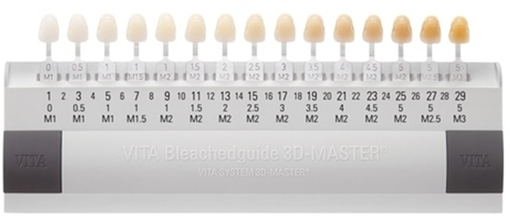


**Figure S2**. VITA® Bleachedguide: shade guide for monitoring bleaching treatments. With its easy-to-read classification of brightness levels.


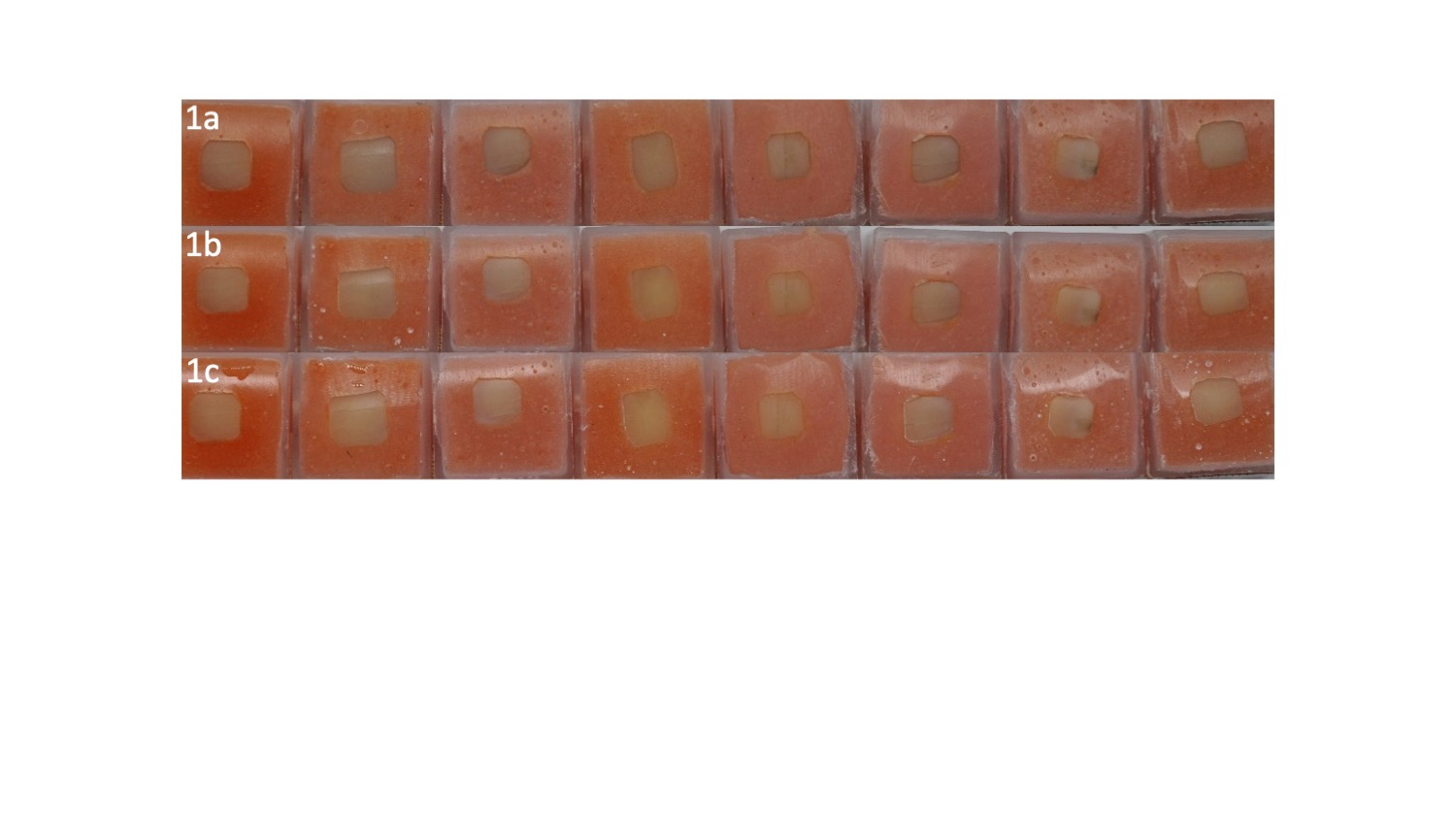


**Figure S3.** Digital photos of enamel samples: 1a) Treatment group 2.1 samples after staining with the coloring solution; 1b) Treatment group 2.1 samples after post-brushing 1 week's worth of brushing; 1c) Treatment group 2.1 samples after post-brushing 1 month's worth of brushing


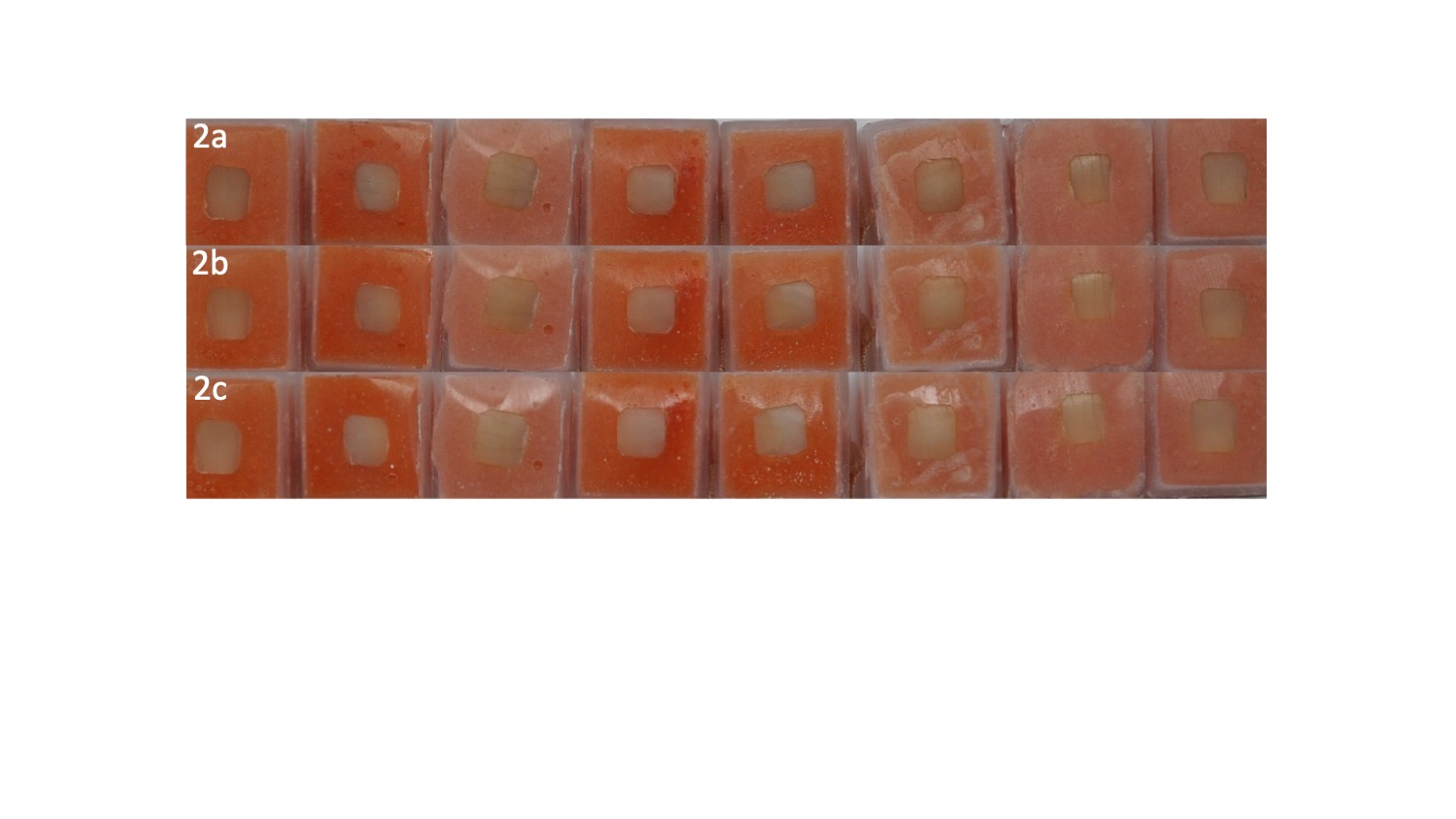


**Figure S4.** Digital photos of enamel samples: 2a) Treatment group 2.2 samples after staining with the coloring solution; 1b) Treatment group 2.2 samples after post-brushing 1 week's worth of brushing; 2c) Treatment group 2.2 samples after post-brushing 1 month's worth of brushing


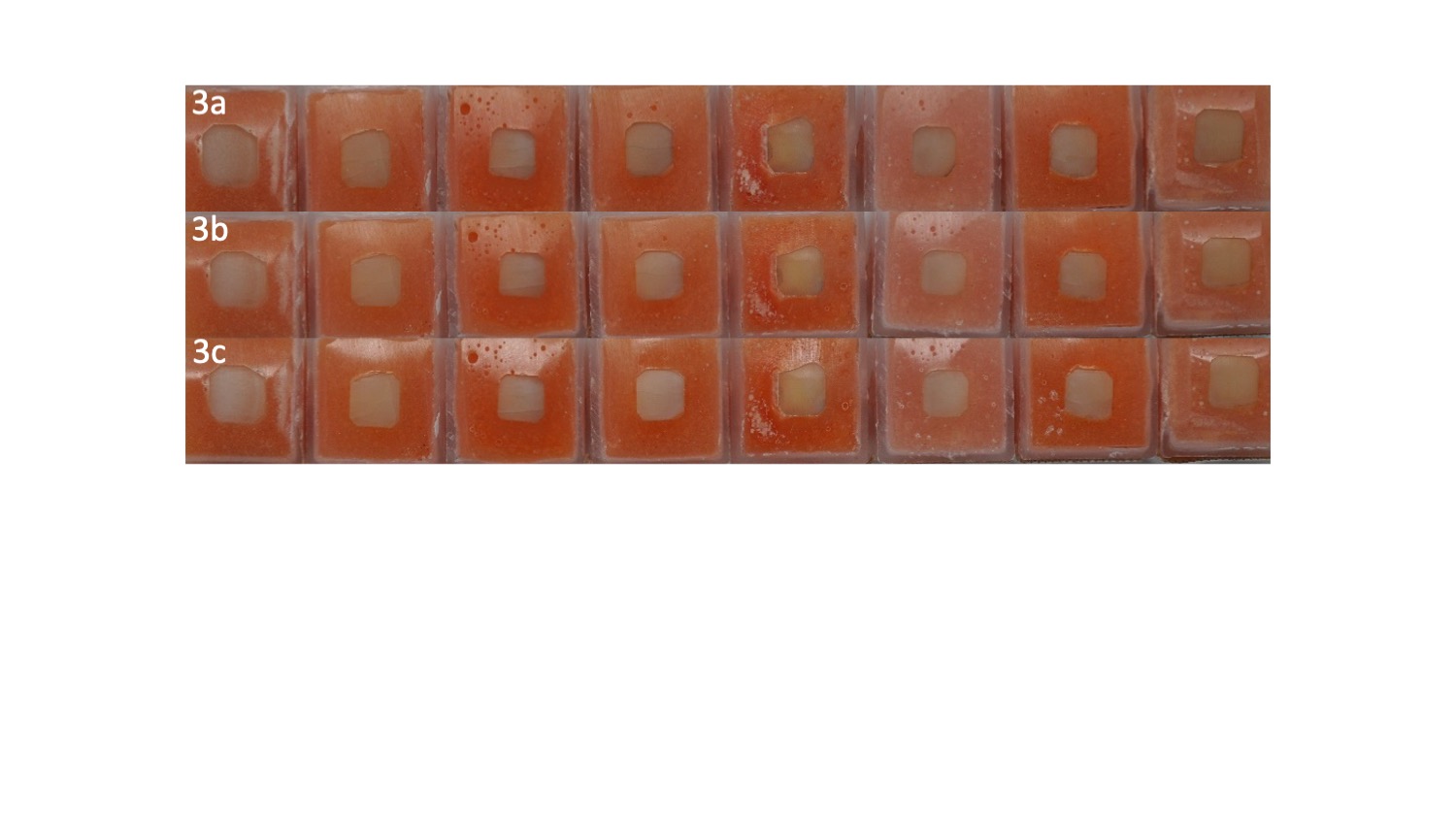


**Figure S5.** Digital photos of enamel samples: 3a) Treatment group 2.3 samples after staining with the coloring solution; 3b) Treatment group 2.3 samples after post-brushing 1 week's worth of brushing; 3c) Treatment group 2.3 samples after post-brushing 1 month's worth of brushing


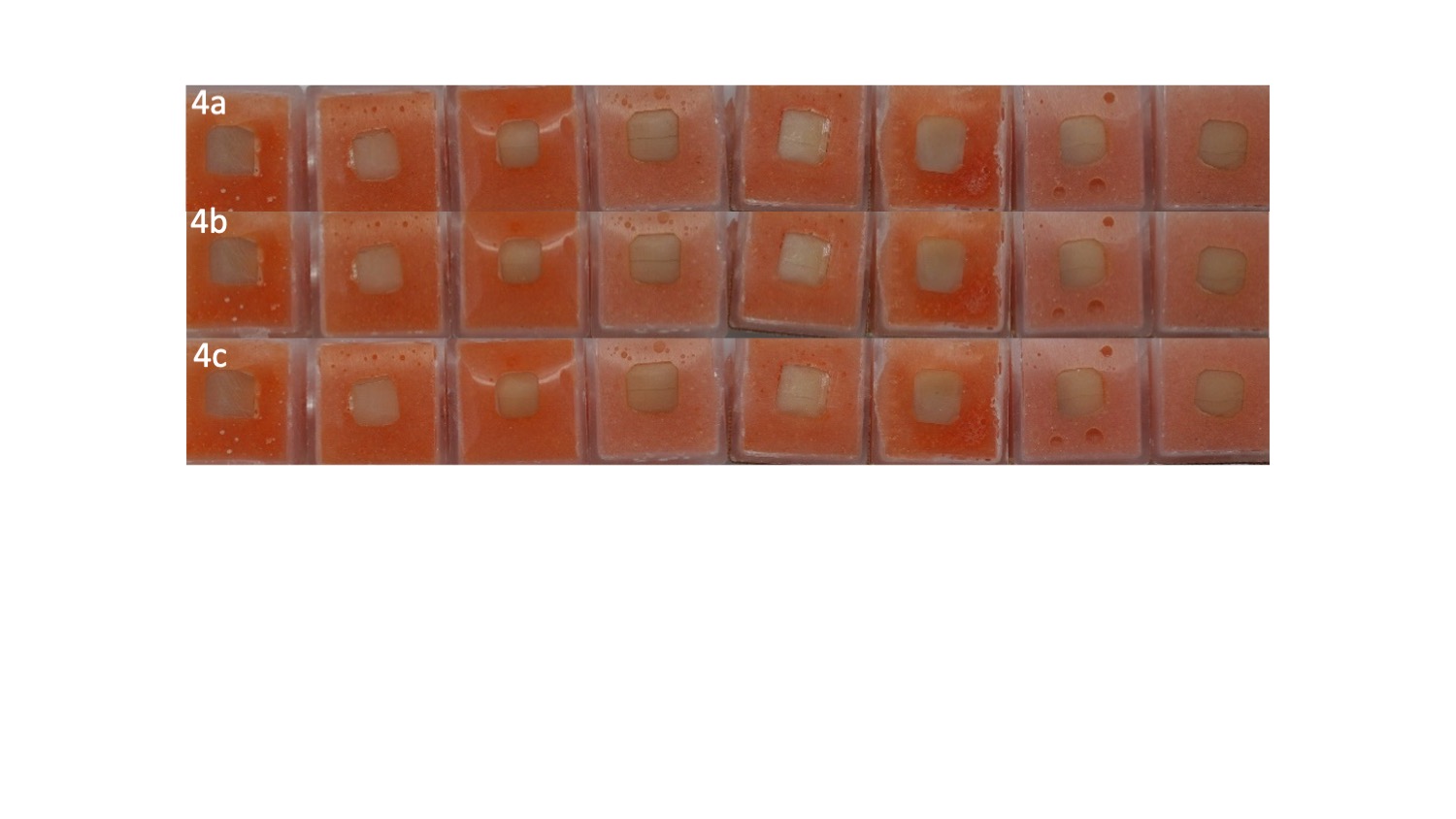


**Figure S6.** Digital photos of enamel samples: 4a) Treatment group 2.4 samples after staining with the coloring solution; 4b) Treatment group 2.4 samples after post-brushing 1 week's worth of brushing; 4c) Treatment group 2.4 samples after post-brushing 1 month's worth of brushing
